# Transplacental Transmission of Porcine Epidemic Diarrhea Virus

**DOI:** 10.3389/fvets.2021.802816

**Published:** 2022-03-15

**Authors:** Jihoon Ryu, Gi-Jong Kang, Onnuri Kim, Jae-Yeon Park, Hyun-Jin Shin

**Affiliations:** ^1^Research Institute of Veterinary Medicine, Daejeon, South Korea; ^2^Laboratory of Infectious Disease, College of Veterinary Medicine, Chungnam National University, Daejeon, South Korea

**Keywords:** porcine epidemic diarrhea virus (PEDV), vertical transmission, transplacental transmission, piglet testicles, umbilical cords

## Abstract

Because the porcine epidemic diarrhea virus (PEDV) is a critical pathogen resulting in rapid spreading and high mortality rates in piglets, understanding of the transmission route of PEDV is required for its controlling. Until now, it is well known that PEDV transmission routes are various, such as fecal–oral route, contaminated feed, farmworkers, and transport vehicles. However, unlike several swine-infected viruses, there were no reports of vertical transmission with PEDV. In our study, we confirmed possible vertical transmission of PEDV. We confirmed PEDV in piglet testicles and umbilical cords from PEDV-positive sow. These findings are direct evidence that PEDV is transmitted vertically through placenta. This is the first report on transplacental transmission of PEDV and will be very important information for controlling PED.

## Introduction

Porcine epidemic diarrhea virus (PEDV) is the etiological agent of porcine epidemic diarrhea (PED), causing dehydration, vomiting, and acute watery diarrhea. Because it has a high mortality rate in neonatal piglets, PEDV is one of the critical pathogens affecting the swine industry (1). PEDV is an enveloped, positive-sense, single-stranded RNA virus that belongs to the genus *Alphacoronavirus*, family Coronaviridae, order Nidovirales. Because PEDV was first discovered in the 1970's, the virus has been widely spread through many countries (2). The direct fecal–oral route of pigs ingesting feces or vomitus from infected pigs is generally considered the major route of PEDV transmission. Furthermore, mechanical transmission, such as on transport vehicles, people entering and working around the farm, and contaminated feed, has also been reported to be major routes of PEDV transmission (1, 3, 4). Alonso et al. (5) reported that PEDV could also display airborne transmission under certain conditions, and Sun et al. (3) and Li et al. (6) suggested that PEDV can be disseminated by transmission in the milk to suckling piglets as another form of oral transmission. We identified many cases of PED-positive piglets and, interestingly, some of them were related to 1-day-old piglets. With this observation, we assumed a possible vertical transmission of PEDV through transplacental route. However, infection via horizontal transmission, such as litter, udder, fence, and other mechanical factors, may have high chance as well. On the basis of our results, there was no compelling evidence of how 1-day-old piglets are infected other than possible vertical transmission; hence, we were interested to investigate it. In this study, we investigated the possibility of PEDV transmission through a transplacental route, which has never been studied in PEDV.

## Materials and Methods

### Sampling

Sows were selected blindly during monitoring period in farms, which have reported as PED-positive cases previously. During the examination period, there was no clinical symptoms associated to PED in either sow or piglets. Moreover, we maintained aseptic conditions to exclude the cross contamination while sampling on farrowing day. As an example, umbilical cords and testicles from each piglet were collected using autoclaved scissors and forceps. Moreover, separate set of instruments, tubes, and bags were used for isolating individual sample. To minimize contamination from environment such as udder and litter, we cleaned as much as possible and collected samples very carefully. A total of 50 testicles (one testicle per piglet) and 13 umbilical cords were collected for this study. The testicles and umbilical cords were washed five times in phosphate-buffered saline (PBS) and frozen at −80°C until use in experiments.

### Reverse Transcription Polymerase Chain Reaction

RT-PCR was carried out for highly conserved PEDV membrane (M) among different PEDV strains. The frozen testicles were transferred in liquid nitrogen and crushed to powder in pestle, and total RNA was extracted by using AllspinTM (GeneAll, Korea). Reverse transcription to cDNA was performed by using SuperiorScript III cDNA Synthesis kit (Enzynomics, Korea) with 1 μg of total RNA. The PEDV M and S1 N-terminal domain (NTD) primer sequences used were as follows: forward: 5′- ATGTCTAACGGTTCTATTCCCG-3′ and reverse: 5′-GACTAAATGAAGCACTTTCTCAC-3′ for PEDV M; and forward: 5′-ATGAAGTCTTTAACCTACTT-3′ and reverse: 5′-AACATATTGCATAGCACAACC-3′ for PEDV S1 NTD. PCR amplification was conducted using 2X TOPsimples DyeMIX-tenuto (Enzynomics) and the following cycling conditions: Denaturation for 10 min at 95°C, 30 cycles of 30 s at 95°C, 30 s at 58°C, and 45 s at 72°C; and a 7-min final extension at 72°C. PCR products were separated at 0.8% agarose gel electrophoresis and visualized using Lumino Graph II (ATTO, Japan).

### Immunohistochemistry

IHC was carried out using testicles that are shown positive or negative results in RT-PCR. The testicle tissues were fixed in formalin, embedded in paraffin, and cut into 3-μm sections. To detect PEDV, slides were deparaffinized in xylene and rehydrated through graded ethanol. Endogenous peroxidase activity in the tissue was blocked with 3% hydrogen peroxide. After blocking with 5% normal goat serum, slides were incubated with homemade anti-PEDV monoclonal antibody as 1:100 dilution in 0.1 ml of SignalStain® Antibody Diluent (Cell Signaling Technology, Beverly, MA). Then, Goat anti-mouse immunoglobulin G (IgG) Alexa Fluor Plus 488 (ThermoFisher Scientific, Waltham, MA) was applied as a secondary antibody at a concentration of 1 μg/ml for 2 h at room temperature. Hoechest 33,342 dye was used for staining nuclei, and slides were mounted in Anti-fade Fluorescence Mounting Medium (Abcam, Cambridge, MA) and then washed. The stained tissues were examined under fluorescence microscope (Optinity KI-2000F; Korea Lab Tech, Korea), and images were analyzed by using Optinity OpticView v3.7 software (Korea Lab Tech).

### Virus Isolation

For virus isolation, Vero cells were seeded into 12-well cell culture plates (1.5 × 10^5^ cells per well). The frozen umbilical cords were homogenized with 10 volumes of pre-chilled serum-free minimum essential media (MEM) containing antibiotics and then centrifuged at 3,000 rpm for 15 min. The supernatants were filtered through 0.45 μm, and Vero cells were infected with 100 μl of tissue homogenates. After initial incubation for 2 h, cells were washed with PBS for three times and replaced with a fresh MEM containing 5 μg/ml of trypsin (BioShop, Canada) and 2% Antibiotic-Antimycotic (Gibco, UK). Blind passages continued until cytopathic effects (CPEs) were observed.

## Results

### Identification of PEDV in Piglet Testicles

**Figure 1:**
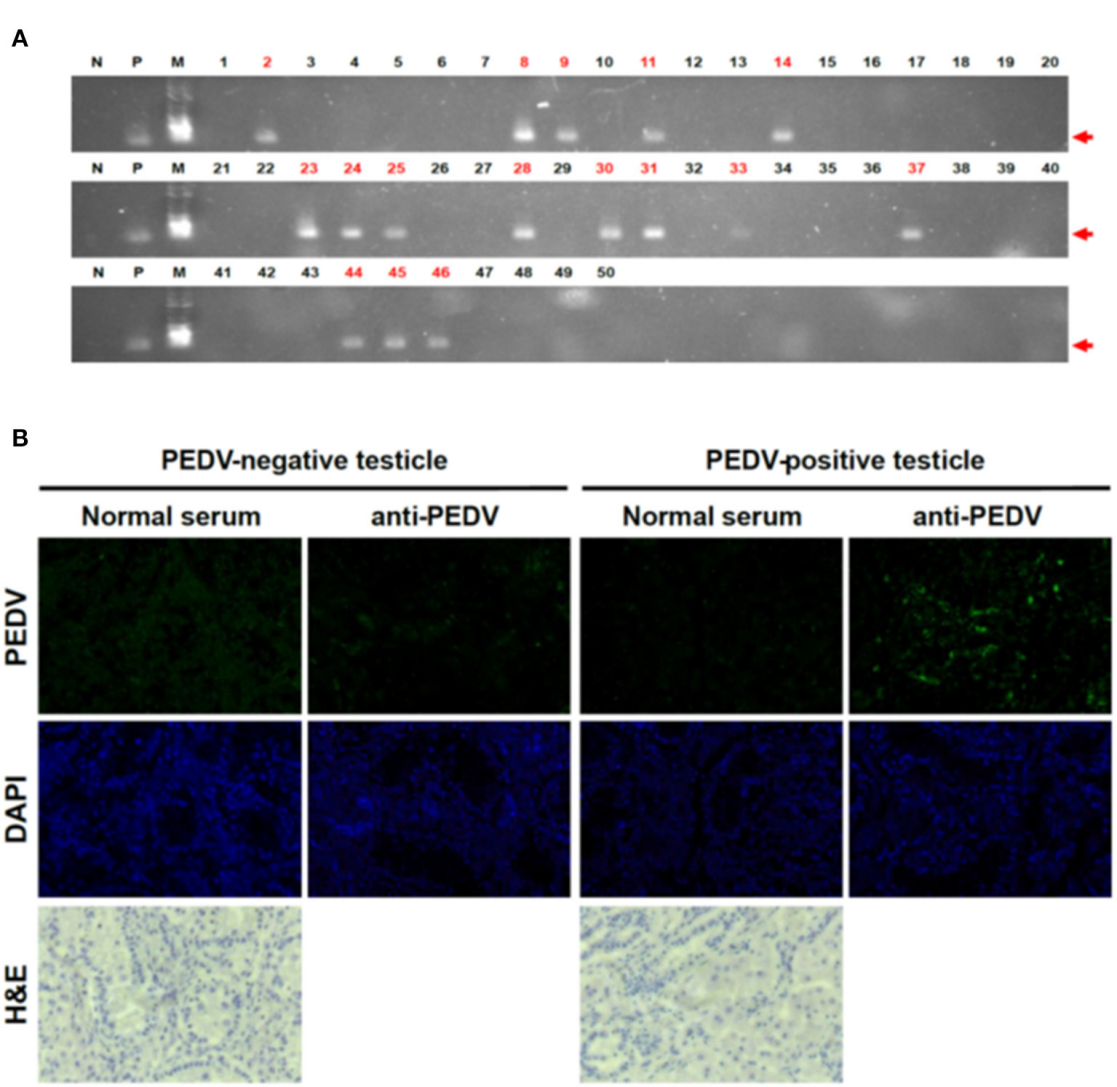
Detection of PEDV in piglet testicles tissues. **(A)** RT-PCR results on testicles of piglets. We performed RT-PCR targeting for PEDV M gene (N, negative control; P, positive control; M, marker). Red numbers indicate positive samples. **(B)** IHC on testicle samples clearly confirmed PEDV positive. We used PEDV-positive sera as the primary antibody and used anti-mouse IgG conjugated with Alexa 488 as the second antibody.

To test the possibility that PEDV can be transmitted by the transplacental route, a total of 50 piglet testicles and 13 umbilical cords were collected on the day of birth, and individual samples were placed in resealable bags to prevent cross-contamination. The testicles and umbilical cords were washed five times in PBS and frozen at −80°C until use in the experiments. To confirm PEDV in piglets born to PEDV-positive sows, RT-PCR was performed, targeting a highly conserved PEDV M gene. The frozen testicles were transferred to liquid nitrogen and crushed to a powder in a pestle, and total RNA extraction, reverse transcription to cDNA, and PCR were performed as described previously (7). We found that 16 of 50 testicle samples tested were positive for PEDV (Figure 1A). As they were collected immediately after birth and had no possible other sources of infection, these PCR-positive results clearly confirmed infection before they were born.

To exclude technical errors by RT-PCR, we reconfirmed the results by IHC with testicles that showed positive or negative RT-PCR results. Consistent with the RT-PCR results, very strong signals were observed in the tissues (Figure 1B). The IHC results clearly confirmed that not only the PEDV genome but also PEDV proteins were present in neonatal piglet testicle samples. These results strongly support our finding that the piglets were PEDV infected before they were born, confirming infection via the placenta.

### Identification of PEDV in Umbilical Cord

Among swine viral diseases, porcine reproductive and respiratory syndrome virus (PRRSV), classical swine fever virus (CSFV), and porcine circovirus (PCV) are representative viruses that show transplacental transmission, during which the viruses are usually found in the umbilical cord, umbilical cord blood, or placenta (8–10). However, there have been no reports on vertical transmission of PEDV. Sun et al. reported that transmission via the milk is one of the possible spreading pathways of PEDV from sows to suckling piglets. However, their results only confirmed possible transmission by sow milk, and no vertical transmission was proven (3, 6). However, to confirm vertical transmission, the virus needs to be shown to be transmitted through the placenta before farrowing, meaning the piglets already have PEDV at the time of birth.

**Figure 2:**
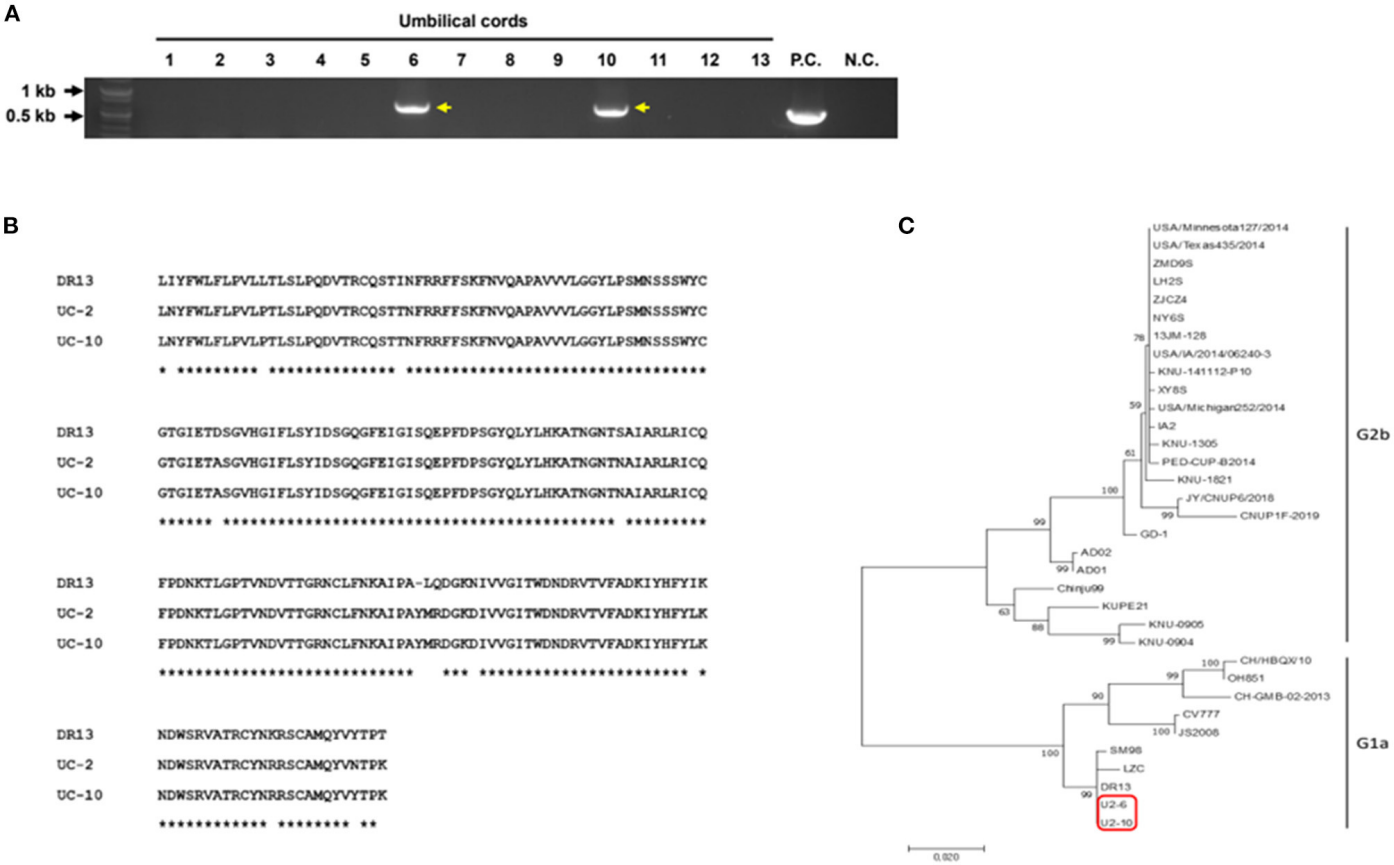
Detection and characterization of PEDV from umbilical cord tissues. **(A)** RT-PCR results on 13 umbilical cords targeting PEDV M gene (P.C., positive control; N.C., negative control). **(B)** Amino acid alignment of the partial PEDV spike protein. Partial PEDV spike was sequenced. * indicates identical sequences. **(C)** Phylogenetic tree of partial PEDV spike genes from published PEDV strains from GenBank and PEDVs from umbilical cords (red box).

In our study, as PEDV was found in neonatal piglet testicles, we speculated that PEDV could be transmitted through the placenta from the sow to the piglets *in utero*. To verify our hypothesis, RT-PCR was performed to detect PEDV in umbilical cord tissues. We found that two of 13 umbilical cord samples were positive (Figure 2A). Because the spike (S) gene is crucial for the characterization and differentiation of PEDVs, RT-PCR was conducted targeting the partial PEDV S1 [621 base pair (bp)] region that is the most variable. The sequences were analyzed using National Center for Biotechnology Information BLAST and compared with several reference sequences of the PEDV S gene in GenBank. Phylogenetic analysis on the basis of partial PEDV S1 was performed using MEGA-X software by the neighbor-joining method with 1,000 bootstrap replicates. The sequence homology results indicated that PEDV S1 from umbilical cords showed nucleotide identities of 94–95% with G1a group strain DR13 (Figure 2B). The phylogenetic tree results strongly confirmed that these PEDVs found in umbilical cords showed high homology with the PEDV G1a strains (Figure 2C).

### Isolation of PEDV From Umbilical Cords

**Figure 3:**
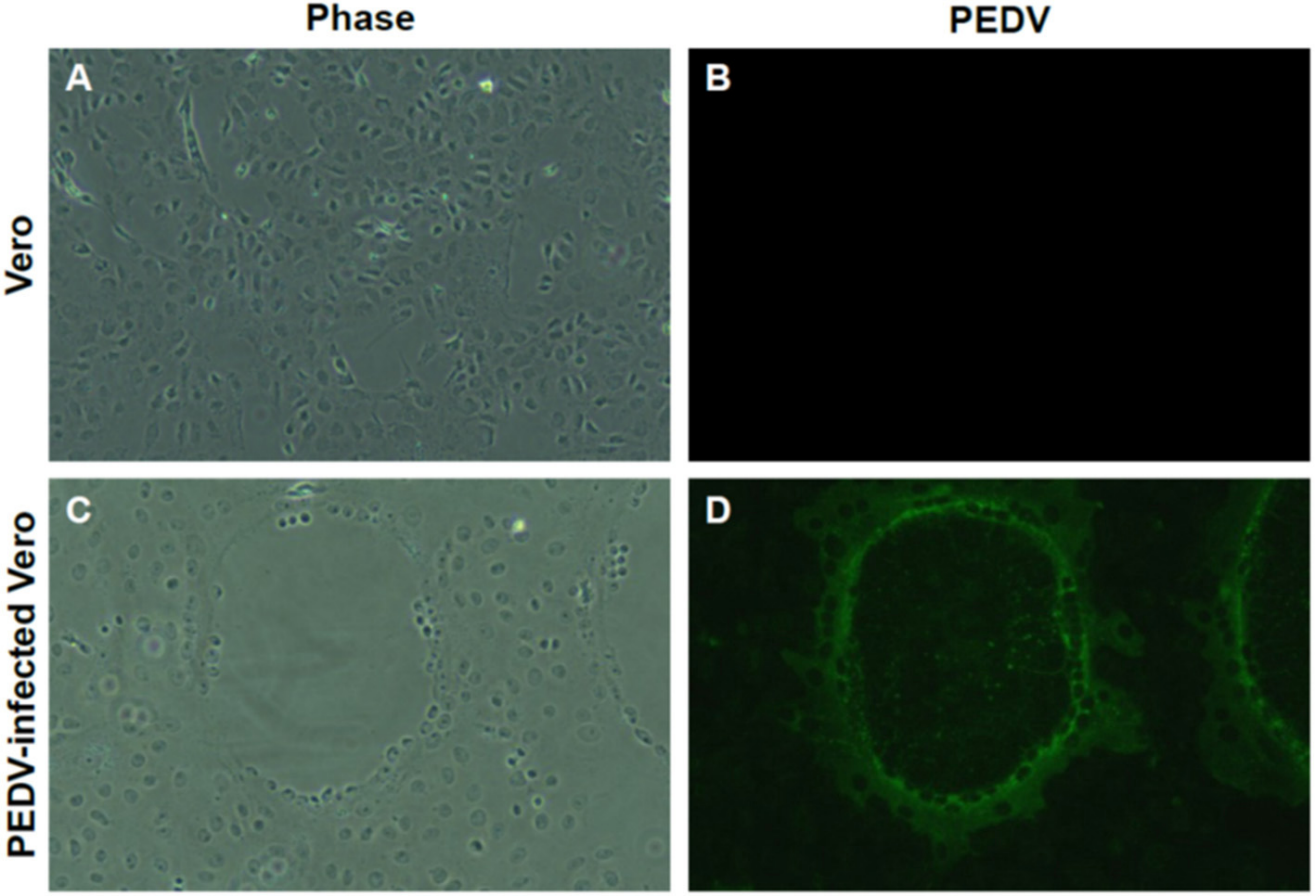
Isolation of PEDV from umbilical cord. **(A,B)** Non-infected Vero cells. **(C)** Cytopathic effect (CPE) formation of newly isolated PEDV from the umbilical cord. Clear CPE formation like cell clumping and fusion was found in Vero cells infected with newly isolated. **(D)** Confirmation of PEDV infection by IFA. Confirmation of PEDV and its replication by IFA using PEDV-positive sera. Magnification, ×400.

RT-PCR–positive samples were proceeded for virus isolation. In addition, we found clear CPE formation by isolated PEDV (Figure 3C), but not in the non-infected Vero cells (Figure 3A). Isolate produced typical and clear CPE, similar with other PEDVs on Vero cells in the laboratory. Isolation was confrimed by Immunofluorescent Assay (IFA) using anti-PEDV antibodies (Figures 3B,D). PEDV antibodies were detected in the same location where CPE was formed (Figure 3D).

## Discussion

Although continuous vaccination programs are ongoing worldwide, at least in Korea, with all pigs being vaccinated every year, there are many PEDV issues that remain unclear. In PED epidemiology studies, repeated outbreaks on the same farms that had previously experienced PED outbreaks are very common. Several transmission methods for PEDV have been reported, and among those, direct contact with infected feces might be the main source of transmission. To date, there have been no reports on vertical transmission of PEDV through the transplacental route.

In this study, we reported direct evidence for transplacental transmission of PEDV by detecting the virus in umbilical cords and day-old piglet testicles. We hypothesized that asymptomatic carrier sows are the main source of PEDV infection because we found many PEDV-positive samples among newborn piglets. Our findings in this study clearly support our hypothesis. Although it has been previously reported that PEDV infection could occur through colostrum or milk, our results clearly confirm that PEDV infection occurred much earlier, before birth. The piglets were infected in the uterus or via the placenta, and the piglets were born with the infection. This is the only way to explain how 1-day-old piglets were infected with PEDV.

In the reference studies, several viral diseases in swine, such as CSFV, porcine parvovirus, PCV, and PRRSV, have been confirmed vertical transmission. Because these viruses mostly cause reproductive disorders like abortion and stillbirth, vertical transmission is considered as a critical spreading route and significant financial impact on the swine industry (9, 11, 12). Possible routes of vertical transmission are either through the placenta, infection during or after birth, or through colostrum. It also well studied that among these vertical transmission routes, transplacental transmission is tightly related to a persistent infection in which viruses are perpetuated in pig herds (13–16). Because of this important reason, a circulating PEDV in the swine farm and irregulated outbreak might be explained by our finding.

However, additional studies are necessary. Our main question now is how the sows acquired PEDV. This could be a very important finding with implications for controlling PEDV outbreaks. Here, we reported, for the first time, the detection of vertical transmission of PEDV. Our data provide novel and very important information about the pathway of PEDV transmission and will be helpful for establishing a vaccine strategy for PEDV.

## Conclusions

The aim of our study is to find evidence on vertical transmission of PEDV. As far as our present knowledge goes, this is the first report to provide scientific results for vertical transmission of PEDV through placenta. We found PEDV in the testicle tissues from piglets and in the umbilical cords from PEDV-positive sow by molecular and immunological analysis. Furthermore, PEDV was successfully isolated from umbilical cords. These results strongly support that PEDV is vertically transmitted from sow to piglets. Despite the need for further studies to complement our results, at least, we found that PEDV spreading is taking place through more diverse routes than we think. In addition, our findings will be helpful in designing a vaccine program to prevent PEDV spreading.

## Data Availability Statement

The datasets presented in this study can be found in online repositories. The names of the repository/repositories and accession number(s) can be found in the article/Supplementary Material.

## Ethics Statement

Ethical review and approval was not required for the animal study because this is studies in the farm and field study.

## Author Contributions

H-JS and JR drafted the manuscript and all authors contributed to the general interpretation of the data and the writing. In addition, H-JS supervised RT-PCR, immunohistochemistry, and sequencing analysis. G-JK and JR harvested testicles and umbilical cords in the swine farm and performed immunohistochemistry and RT-PCR. OK and J-YP performed immunohistochemistry and sequencing analysis. All authors read and approved the final manuscript.

## Funding

This research was supported by a grant from the Technology Development Program for Agriculture and Forestry, Ministry for Food, Agriculture, Forestry and Fisheries, Republic of Korea.

## Conflict of Interest

The authors declare that the research was conducted in the absence of any commercial or financial relationships that could be construed as a potential conflict of interest.

## Publisher's Note

All claims expressed in this article are solely those of the authors and do not necessarily represent those of their affiliated organizations, or those of the publisher, the editors and the reviewers. Any product that may be evaluated in this article, or claim that may be made by its manufacturer, is not guaranteed or endorsed by the publisher.
